# A Rare Case of Myopericarditis Following Campylobacter Gastroenteritis in an Immunocompetent Young Adult

**DOI:** 10.7759/cureus.83224

**Published:** 2025-04-29

**Authors:** Konstantinos Baronos, Huili James Chong, Lavanya Athithan, Subrahmanya Srinivas Varanasi

**Affiliations:** 1 Medicine, University Hospitals of Leicester NHS Trust, Leicester, GBR; 2 Cardiology, University Hospitals of Leicester NHS Trust, Leicester, GBR

**Keywords:** acute myopericarditis, bacterial pericarditis, campylobacter enteritis myocarditis, campylobacter gastroenteritis, successfully treated myopericarditis

## Abstract

Myopericarditis is a rare but recognised extra-intestinal manifestation of *Campylobacter jejuni *infection, particularly in the absence of any underlying disease. While *Campylobacter *is a common cause of bacterial gastroenteritis, its potential to cause cardiac involvement is usually underestimated.The pathogenic process is not well elucidated but is most likely to involve direct bacterial invasion, immune processes, or systemic inflammation. In the early stages, symptoms may simulate acute coronary syndromes and therefore present diagnostic challenges. We present the case of a previously healthy young adult patient who had developed myopericarditis following an episode of self-limiting *Campylobacter *gastroenteritis. The diagnosis was established by electrocardiography, cardiac biomarker testing, echocardiogram (ECG), and cardiac magnetic resonance imaging (CMRI). Late gadolinium enhancement revealed sub-epicardial enhancement in the basal inferior and inferolateral segments, consistent with myopericarditis. The patient was conservatively managed with intravenous fluids, analgesia, and cardiac monitoring and made a complete recovery. This case emphasises the requirement of a strong suspicion of cardiac complications in patients presenting with chest pain following recent gastrointestinal illness. Prompt identification and adequate management can yield excellent results and prevent disastrous outcomes.

## Introduction

*Campylobacter *is one of the most common causes of bacterial gastroenteritis globally. This Gram-negative microorganism is typically transmitted via the oral-faecal route through the consumption of unpasteurised milk, undercooked meat, and untreated water [1]. The majority of these cases are self-limiting; however, they can present as extraintestinal manifestations such as Guillain-Barré syndrome, Miller Fisher syndrome, bacteremia, meningitis, reactive arthritis, and myocarditis [2].

Myocarditis is defined as the inflammation of the myocardium with a wide array of causes. Viruses are the main infectious agents responsible for myocarditis; the relevant implicated viruses are adenovirus, parvovirus B19, human herpesvirus 6, adenovirus, and enteroviruses [3, 4]. Cardiac complications of *Campylobacter jejuni *are extremely rare, but there appears to be an increasing recognition of this sequela. The only reported incidence in the literature comes from a Danish cohort study in 2007 that reported 16.1 per 100,000 person-years [5].

The exact pathophysiology of *Campylobacter*-associated myocarditis still remains unknown, although there are theories of direct bacterial invasion of the myocardium, damage from circulating toxins, and an immune-mediated process [6, 7]. The autoimmune theory is hypothesised to be due to reinfection with *Campylobacter jejuni* due to sensitisation from prior exposure [8]. Currently, there is no standardised treatment for *Campylobacter*-associated myocarditis. Management is primarily supportive, typically including analgesia and intravenous fluids; however, macrolide antibiotics have been used in some cases [9]. In severe cases of decompensated heart failure, the implementation of the four foundational pillars of heart failure therapy is essential. These include beta-adrenergic receptor blockers (beta-blockers), angiotensin receptor-neprilysin inhibitors (ARNIs) or angiotensin-converting enzyme (ACE) inhibitors/angiotensin II receptor blockers (ARBs), mineralocorticoid receptor antagonists (MRAs), and sodium-glucose co-transporter 2 inhibitors (SGLT2) [7].

In this report, we present a rare case of myopericarditis following *Campylobacter* enteritis in an immunocompetent young adult.

## Case presentation

A 21-year-old Caucasian male university student with no significant past medical history presented with a two-day history of intermittent watery diarrhoea, vomiting, and subsequent oral fluid intolerance. He had no history of recent travel, consumption of contaminated food or water or recent viral infections. One day after the onset of gastrointestinal symptoms, he developed severe, persistent, sharp left-sided chest pain without radiation, accompanied by epigastric discomfort and dark urine. He had no history of cardiovascular disease, recent trauma, or illicit drug use.

On examination, the patient was alert and orientated, with a Glasgow Coma Scale (GCS) of 15/15, afebrile, and appeared clinically dehydrated. Respiratory examination revealed clear chest auscultation bilaterally. Cardiovascular examination demonstrated normal heart sounds, no added sounds, murmurs or pericardial rub, and symmetrical, palpable peripheral pulses. There was no peripheral oedema. Abdominal examination revealed a soft abdomen with epigastric tenderness but no organomegaly; bowel sounds were present.

**Figure 1:**
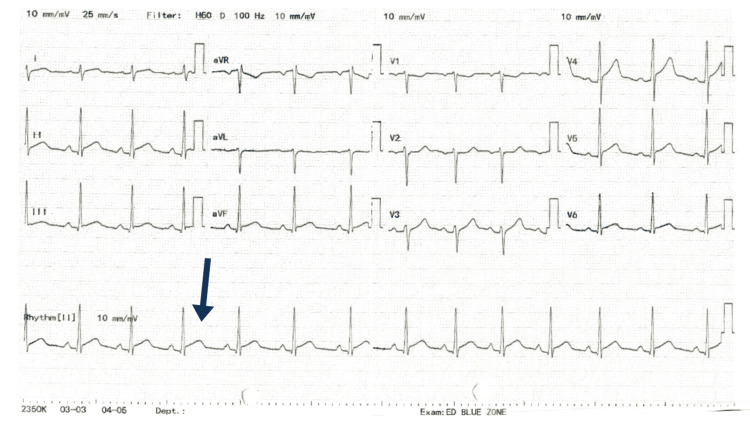
12-Lead Electrocardiogram (ECG) Showing Widespread ST-Segment Elevation in Leads II, III, aVF, V4, V5, and V6. Lead II (Highlighted by an Arrow) Shows Significant ST Elevation in One of the Cardiac Cycles, Which Is a Key Finding in This Case.

Initial investigations included an electrocardiogram (ECG), which showed global ST-segment elevation with sinus rhythm (Figure 1). Cardiac biomarkers were markedly elevated, with high sensitivity troponin levels peaking at 6,275 ng/L on admission, before decreasing to 3,915 ng/L 3 hours later. Inflammatory markers were elevated, including a raised C-reactive protein (CRP) and white cell count with neutrophilia (Table 1). Extended respiratory polymerase chain reaction (PCR) panels excluded viral pathogens (SARS-CoV-2, influenza, and respiratory syncytial virus (RSV)).

**Table 1:** Summary of Key Blood Test Results Obtained on Hospital Admission

Full Blood Count (FBC)	Value	Unit	Reference Range
White Cell Count	12.1	x10^9/L	4.0 - 11.0
Red Blood Count	5.46	x10^12/L	4.50 - 6.50
Haemoglobin	156	g/L	130 - 180
Haematocrit	0.449	L/L	0.400 - 0.540
Mean Cell Volume	82	fL	80.0 - 99.0
Mean Cell Haemoglobin	28.6	pg	27.0 - 32.0
Platelet Count	234	x10^9/L	140 - 400
Neutrophil Count	8	x10^9/L	1.50 - 7.50
Total Lymphocyte Count	2.65	x10^9/L	1.00 - 4.00
Monocyte Count	1.28	x10^9/L	0.20 - 0.80
Eosinophil Count	0.15	x10^9/L	0.04 - 0.40
Basophil Count	0.04	x10^9/L	0.02 - 0.10
Nucleated Red Blood Cells	<0.20	x10^9/L	0.00 - 0.20
Sodium	136	mmol/L	133 - 146
Potassium	3.5	mmol/L	3.5 - 5.3
Urea	2.8	mmol/L	2.5 - 7.8
Creatinine	74	umol/L	60 - 120
Estimated Glomerular Filtration Rate (eGFR)	>90	mL/min/1.73m2	
International Normalised Ratio (INR)	1.2		
Prothrombin Time	16	Secs	12.0 - 15.0
Activated Partial Prothrombin Time (APTT)	36.7	Secs	26.1 - 36.6
C-Reactive Protein	84	mg/L	0 - 10
Total Protein	79	g/L	57 - 82
Albumin	50	g/l	35 - 50
Alkaline Phosphatase (ALP)	72	U/L	30 - 130
Alanine Transaminase (ALT)	24	U/L	10 - 49
Total Bilirubin	54	umol/L	0 - 21.0
Amylase	23	U/L	30 - 118
Troponin I	6275	ng/L	<0.04

**Figure 2:**
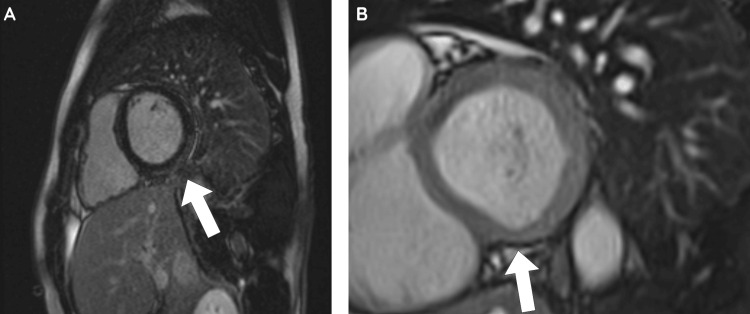
Cardiac Magnetic Resonance Imaging (CMRI), Late Gadolinium Enhancement (LGE) Imaging, and Short-Axis Cross-Section. Image A Shows Late Gadolinium Subepicardial Enhancement in the Basal Inferior and Inferolateral Segments (Highlighted by the Arrow). Image B Shows a Still Image of the Mild Hypokinesis of the Basal Inferior and Inferolateral Segments (Highlighted by the Arrow).

**Figure 3:**
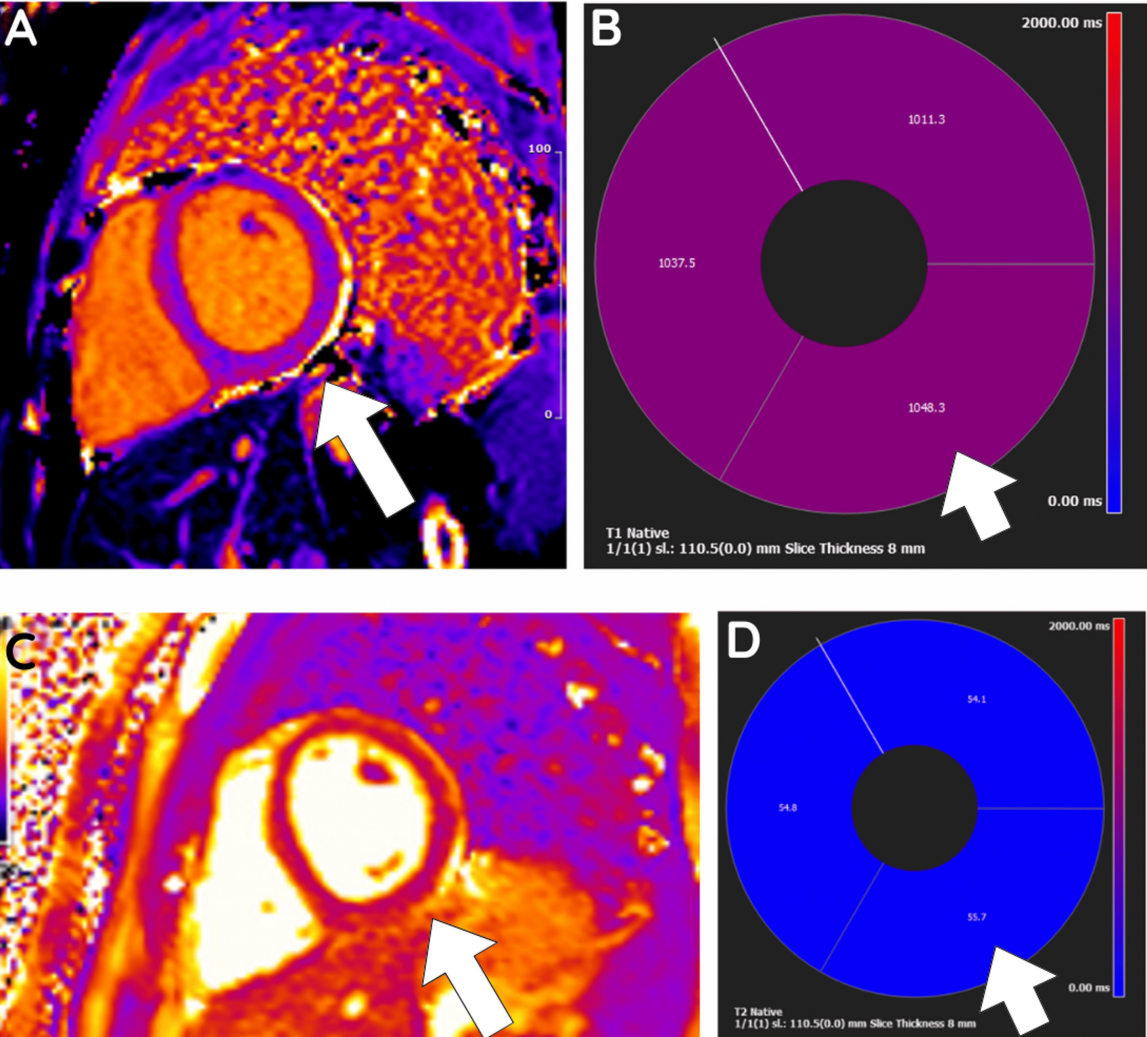
T2 Map on Cardiac Magnetic Resonance Imaging (CMRI) Image A Is a Cross-Section of the Basal Segment Showing Increased T1 in the Basal Inferior and Inferoseptal Segments. Image B Shows Superimposition of Myocardial Segments With Corresponding T1 Times. Image C Is a Cross-Section of the Basal Segment Showing Increased T2 Relaxation Times Throughout but Most Pronounced in the Basal Inferior and Inferolateral Segments. Image D Is a Superimposed Map of the T2 Times on Average in the Sections Seen.

Transthoracic echocardiography (TTE) was performed to further evaluate these findings and showed good biventricular systolic function, with a left ventricular ejection fraction (LVEF) of 55% to 60% with no regional wall motion abnormalities. The pericardium appeared bright in the inferior/inferolateral regions, but no pericardial effusion was present. Cardiac imaging was performed to further delineate this in the presence of the clinical findings and investigations above. Cardiac magnetic resonance imaging (CMRI) demonstrated preserved biventricular systolic function, with an LVEF of 53%. Mild hypokinesis of the basal inferior and inferolateral segments was noted. Late gadolinium enhancement revealed sub-epicardial enhancement in these regions, consistent with myopericarditis (Figure 2). Native T1 and T2 values of the relevant segment were elevated in comparison to the septum (Figure 3). No significant valvular abnormalities or pericardial effusion were observed. Faecal culture, which was collected on admission and sent to the lab, returned after three days with a positive culture for *Campylobacter *species.

The patient was conservatively managed with intravenous fluids, analgesia, and cardiac monitoring. His symptoms completely resolved within three days, and follow-up bloods and ECG were normal. The patient was then discharged home and advised to avoid heavy exercise for one month and to have a cardiology follow-up appointment to evaluate if there were any residual symptoms. Imaging and clinical presentation were consistent with a diagnosis of acute myopericarditis, which was most likely of bacterial origin, considering the preceding gastrointestinal symptoms.

## Discussion

This case represents an unusual but increasingly recognised complication of *Campylobacter* infection - myopericarditis. *Campylobacter* *jejuni* is a common cause of bacterial gastroenteritis, but its ability to cause extraintestinal manifestations such as neurological, musculoskeletal, and cardiac complications is underrecognised. *Campylobacter*-associated myocarditis is rare. Epidemiological data illustrate its rarity, with an incidence of 16.1 cases per 100,000 person-years reported in *Campylobacter*-infected individuals [2]. Although rare, cardiac complications are life-threatening and therefore diagnosis requires a high index of suspicion from the clinician.

The pathophysiology of myopericarditis with *Campylobacter* infection remains poorly understood. Histopathological findings in a fatal case demonstrated focal myocytolysis, polymorphonuclear infiltration, and a negative PCR test for *Campylobacter* in cardiac tissue. These findings suggest toxin-induced damage rather than primary bacterial invasion [10]. Other theories propose direct bacterial invasion or a cytotoxic T-cell-mediated immunologic reaction [11]. In our patient, the short interval between the presentation of gastrointestinal symptoms and cardiac presentation suggests a direct bacterial invasion or toxin mechanism rather than a delayed immune-mediated process [12]. Hormonal and genetic factors also play a role with the reported male predominance; experimental evidence within viral myocarditis has shown testosterone suppresses anti-inflammatory pathways, quite possibly enhancing susceptibility [13].

The diagnosis of bacterial or viral myocarditis relies heavily on clinical presentation, including chest pain, dyspnoea, significantly elevated cardiac biomarkers and imaging evidence. In this patient, the presence of prominent ST-segment elevations on ECG and subepicardial late gadolinium enhancement on CMRI indicated an acute inflammatory myocardial process. CMRI is an important noninvasive diagnostic method for myocarditis and is a reliable method of characterising the myocardium compared to endomyocardial biopsy due to limitations such as being invasive, infrequent clinical use and overall low sensitivity [14]. The Lake Louise Criteria is currently the agreed consensus used to diagnose myocarditis on CMRI based on the presence of oedema, hyperaemia, and scar/fibrosis [15].

Treatment of *Campylobacter*-associated myopericarditis is primarily supportive. Initial therapy includes intravenous fluids, analgesia, and close cardiac monitoring. Correcting electrolyte imbalances is important to prevent potentially lethal arrhythmias. In the context of complicating cardiogenic shock, vigorous support, including extracorporeal membrane oxygenation (ECMO), may be required, as outlined in reviews of fulminant myocarditis [16]. Although macrolides or fluoroquinolones are effective in the management of gastrointestinal *Campylobacter* infection, their role in modulating myocardial inflammation is uncertain [9]. Additionally, while colchicine reduces pericardial inflammation in acute pericarditis (as demonstrated in a randomised controlled trial of recurrent pericarditis [17]), its routine use in *Campylobacter*-associated myopericarditis is unestablished.

The prognosis for *Campylobacter* myocarditis is generally good, and the majority of patients recover completely. Patients with myocarditis can, however, proceed to develop dilated cardiomyopathy, ongoing arrhythmia, or cardiogenic shock in extreme cases [18]. Serial echocardiographic and CMRI follow-up scans are used at the discretion of the treating clinician based on the presence of higher-risk features, including the degree of LV dysfunction and fibrosis. This helps assess recovery as well as providing recommendations for activity, which ideally should be restricted for a duration of at least four to six weeks from the acute attack.

## Conclusions

This article emphasises the importance of clinical suspicion of atypical but severe complications of common gastrointestinal infections such as *Campylobacter *infection*. *While they are mostly self-limiting and can be managed conservatively, extra-intestinal complications like myopericarditis may pose diagnostic and therapeutic dilemmas, especially in young patients without any associated comorbidities. Overlap in presentation with more common cardiac conditions, such as myocardial infarction, makes it essential to have a thorough assessment with ECG, biomarkers, and imaging. Early exclusion and detection of other life-threatening illnesses are crucial to avoid unnecessary interventions and administer appropriate therapy. This paper contributes to the sparse literature base on *Campylobacter*-associated myopericarditis and reiterates the importance of making a broad differential diagnosis in patients presenting with recent gastrointestinal disease and chest pain. Further studies are needed to define optimal therapy, long-term outcomes, and whether bacterial virulence or host factors drive severity.

## References

[1] Graham A, Hawkins L, Balasegaram S, Narasimhan S, Wain J, Clarke J, Manuel R (2024). A decade of Campylobacter and Campylobacter bacteraemias in a district general hospital and the surrounding London and South East region, England. J Infect.

[2] Kaakoush NO, Castaño-Rodríguez N, Mitchell HM, Man SM (2015). Global epidemiology of Campylobacter infection. Clin Microbiol Rev.

[3] Lampejo T, Durkin SM, Bhatt N, Guttmann O (2021). Acute myocarditis: aetiology, diagnosis and management. Clin Med (Lond).

[4] Schultheiss HP, Baumeier C, Aleshcheva G, Bock CT, Escher F (2021). Viral myocarditis-from pathophysiology to treatment. J Clin Med.

[5] Becker S, Ejlertsen T, Kristensen B, Nørgaard M, Nielsen H (2007). Is the incidence of perimyocarditis increased following Campylobacter jejuni infection?. Eur J Clin Microbiol Infect Dis.

[6] Suehiro W, Nishio R, Noiri JI (2023). Acute myocarditis secondary to Campylobacter jejuni enteritis: a case report. J Cardiol Cases.

[7] De Cock D, Hiltrop N, Timmermans P, Dymarkowski S, Van Cleemput J (2012). Myocarditis associated with Campylobacter enteritis: report of three cases. Circ Heart Fail.

[8] Yaita S, Tago M, Hisata Y, Fujiwara M, Yamashita SI (2020). Relapse of acute myocarditis associated with Campylobacter jejuni enterocolitis. Clin Case Rep.

[9] Hessulf F, Ljungberg J, Johansson PA, Lindgren M, Engdahl J (2016). Campylobacter jejuni-associated perimyocarditis: two case reports and review of the literature. BMC Infect Dis.

[10] Pena LA, Fishbein MC (2007). Fatal myocarditis related to Campylobacter jejuni infection: a case report. Cardiovasc Pathol.

[11] Chantzaras AP, Karageorgos S, Panagiotou P (2021). Myocarditis in a pediatric patient with Campylobacter enteritis: a case report and literature review. Trop Med Infect Dis.

[12] Shastri A, Al Aiyan A, Kishore U, Farrugia ME (2023). Immune-mediated neuropathies: pathophysiology and management. Int J Mol Sci.

[13] Feldman AM, McNamara D (2000). Myocarditis. N Engl J Med.

[14] Polte CL, Bobbio E, Bollano E (2022). Cardiovascular magnetic resonance in myocarditis. Diagnostics (Basel).

[15] Ferreira VM, Schulz-Menger J, Holmvang G (2018). Cardiovascular magnetic resonance in nonischemic myocardial inflammation: expert recommendations. J Am Coll Cardiol.

[16] Cooper LT Jr (2009). Myocarditis. N Engl J Med.

[17] Imazio M, Brucato A, Cemin R (2011). Colchicine for recurrent pericarditis (CORP): a randomized trial. Ann Intern Med.

[18] Kindermann I, Barth C, Mahfoud F (2012). Update on myocarditis. J Am Coll Cardiol.

